# Longitudinal Single‐Axon‐Resolution Imaging of Peripheral Nerve Injury Response in Mice Using an Optical Window Implant

**DOI:** 10.1002/advs.76375

**Published:** 2026-06-28

**Authors:** Igor D. Luzhansky, Emma Anisman, Ron Perez, Sophia Zhang, Morgan Hoffman, Daniel Hunter, Abigail Cherian, Jevon Bonner, Emilia Feria, Ahmed Ahmed, Muneeb Malik, Junwei Du, Leland C. Sudlow, David M. Brogan, Matthew D. Wood, Mikhail Y. Berezin

**Affiliations:** ^1^ Institute of Materials Science and Engineering Washington University in Saint Louis St. Louis USA; ^2^ Department of Radiology School of Medicine Washington University in Saint Louis St. Louis USA; ^3^ Department of Orthopaedic Surgery School of Medicine Washington University in Saint Louis St. Louis USA; ^4^ Department of Surgery School of Medicine Washington University in Saint Louis St. Louis USA

**Keywords:** axonal degeneration, intravital microscopy, live animal imaging, multimodal imaging, PDMS window, peripheral nerve regeneration, Schwann cells, sciatic nerve, second‐harmonic generation (SHG), two‐photon (2P) microscopy

## Abstract

Chronic, high‐resolution imaging of peripheral nerve injury and regeneration remains a major technical challenge due to the optical inaccessibility of peripheral nerves embedded in soft tissue. Here, we present a broadly applicable approach for longitudinal, high‐resolution optical imaging of peripheral nerves in vivo that combines a flexible, optically transparent polydimethylsiloxane (PDMS) window along with multimodal microscopy. The PDMS window is implanted in the skin above the mouse sciatic nerve, providing stable, motion‐tolerant optical access for over one month without impairing nerve function. Using confocal, two‐photon, and second‐harmonic generation microscopy, we simultaneously visualized fluorescently labeled axons and Schwann cells, endogenous collagen, and vasculature with high spatial and temporal resolution. This approach enabled real‐time tracking of axonal degeneration and regeneration, Schwann cell dynamics, and extracellular matrix‐guided axon pathfinding across multiple nerve injury models. The platform also supports longitudinal imaging of axonal regrowth within a clinically relevant model of nerve transection gap repair. Overall, our method enables new avenues for studying peripheral nerve biology and testing regenerative therapies in vivo.

## Graphical abstract

A minimally invasive optical window enables stable, long‐term imaging of peripheral nerves in vivo at single‐axon resolution. Dynamic processes of degeneration, regeneration, and cellular remodeling are visualized across multiple time scales within the same nerve region, providing a unique platform to study a variety of anatomical and molecular events in the peripheral nerves *Created in BioRender. Berezin, M. (2026)* https://BioRender.com/z6ugwh9.

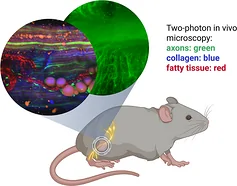

## 1 Introduction

Peripheral nerve injury (PNI) presents a significant clinical challenge, affecting over 100 000 individuals annually in the United States alone [1]. PNI disrupts sensory, motor, and autonomic functions and can lead to long‐term disability. Understanding the cellular and subcellular mechanisms of nerve degeneration and regeneration is essential for developing new therapeutic strategies. Deep imaging methods like MRI and ultrasound currently do not have enough spatial resolution to visualize small anatomical features and individual axons, and they have fewer varieties of contrast agents for labeling specific types of cells and tissue components. Optical methods yield lower resolution at greater imaging depth because of tissue scattering from skin and internal organs, although topically applied high‐absorption dyes can significantly reduce skin scattering [2]. High‐resolution, longitudinal imaging of peripheral nerves in vivo over extended periods has remained elusive due to anatomical and mechanical constraints.

Tissue‐embedded optical windows can resolve some of the resolution challenges from greater imaging depth and have been successful in long‐term imaging of the brain [3, 4] and spinal cord [5, 6]. However, they are limited in their application to peripheral nerves by the absence of stable anatomical support and further risk of tissue injury. Unlike the brain and spinal cord, where optical access using implanted windows is stabilized by bone, peripheral nerves are often embedded in soft and highly mobile muscle and connective tissue and naturally undergo significant mechanical strain due to limb motion. This mobility, coupled with skin and muscle overlay, has limited the development of longitudinal optical imaging platforms for peripheral nerve dynamics. Furthermore, attempts to maintain chronic peripheral nerve implants in mammals have also been hindered by implant instability [7], fibrotic tissue formation [8] and risk of interference with nerve function.

Here, we show an imaging platform that enables chronic, longitudinal, single‐axon‐resolution imaging of a major peripheral nerve in a mammalian model. Our technique combines surgical splitting of the biceps femoris and implantation of a soft, optically transparent, polydimethylsiloxane (PDMS) window secured to the skin over the sciatic nerve. The flexible window accommodates tissue movement while maintaining optical clarity and long‐term stability. Importantly, the method does not interfere with nerve function, as demonstrated by behavioral testing, electrophysiology, and histology.

Longitudinal, in vivo imaging of peripheral nerves provides critical advantages in tracking dynamic cellular responses compared to conventional histological analysis at discrete time points. By enabling continuous observation of the same nerve region within the same animal over time, it eliminates inter‐animal variability as a confounding factor in the interpretation of dynamic cellular events. This makes it possible to directly observe transient events such as Schwann cell (SC) reorganization, axonal fragmentation, and early regenerative sprouting that may otherwise be missed by static tissue sampling at discrete timepoints. It also allows regenerative outcomes to be linked to earlier injury responses within the same subject, providing a more complete picture of peripheral nerve repair over time.

We utilized this approach in transgenic mouse models with fluorescently labeled neurons (Thy1‐YFP [9] and Thy1‐GCaMP [10]) and SCs (S100‐GFP) [11] combined with the use of exogenous markers like Nile Red for myelin labeling [12] and fluorophore‐conjugated tomato lectin for labeling of vascular endothelial cells and some blood cells. The resulting system achieved high‐resolution, multi‐channel, in vivo imaging of peripheral nerve ultrastructure. Further integration of confocal, two‐photon (2P), and second‐harmonic generation (SHG) microscopy facilitated visualization of axons, myelin, SCs, collagen, blood vessels, and endoneurial channels at cellular and subcellular resolution.

Our technique allowed real‐time tracking of axonal degeneration and regeneration across multiple nerve injury models, including partial crush, compression, and transection with conduit repair. We observed SC dynamics within hours of injury, axonal fragmentation by day 2, and the emergence of growth cone‐like structures and regenerating axons by day 4. Regenerating axons were aligned with preserved endoneurial channels, supporting the idea that extracellular matrix (ECM) architecture provides guidance cues for regrowth. Having enabled repeatable, stable, and high‐resolution imaging of the same nerve region over weeks, this platform offers a powerful tool for characterizing dynamic cellular responses to nerve injury and regenerative repair in vivo.

## 2 Results

### 2.1 Hindlimb Skin‐Embedded PDMS Window Enables Long‐Term Sciatic Nerve Imaging

To expose the sciatic nerve without manipulating it directly, we split the overlying biceps femoris muscle (Figure 1a) and installed a PDMS window implant [13] to close the skin incision (Figure 1b). Following recovery from window installation without applied nerve injury, mice appeared clinically normal and walked without apparent asymmetry. To assess retention of PDMS windows, they were implanted in C57Bl6 (Thy1‐YFP, Thy1‐GCaMP, and wild‐type) and B6D2 (S100‐GFP) mice (see details in Table S1). The PDMS window remained securely embedded in the skin for a median of about 7 weeks (Figure 1e), with 90% lasting at least 18 days and 12% for 80 days or more, with a similar trend for all strains. For comparison, rigid 3D‐printed acrylic implants of approximately the same size (Figure S1a,b) had a median retention of about 9 days (Figure 1e).

**FIGURE 1:**
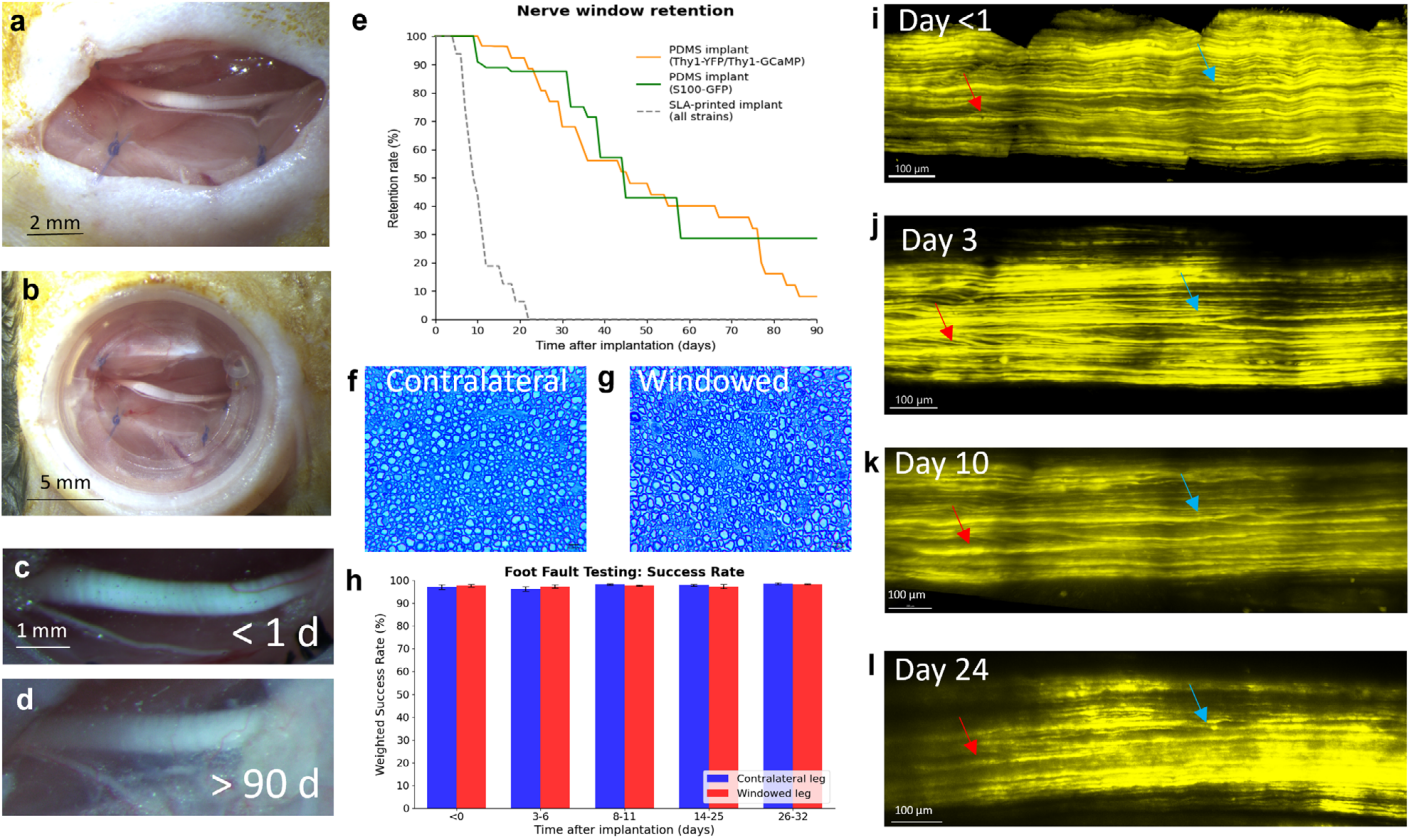
Overview of the mouse sciatic nerve window technique. (a) Representative image of an exposed sciatic nerve during window implantation procedure following skin incision and sutured‐reinforced displacement of the biceps femoris. (b) Sciatic nerve after the window installation. Representative images of a nerve behind the window (c) shortly after window implantation, and (d) after 90+ days with fibrotic tissue removed. (e) Retention rate of different nerve windows (see the description in Table S1). (f,g) Transverse section of the windowed sciatic nerve after 90 days shows normal nerve histology similar to contralateral sciatic nerve section. (h) Foot fault testing of windowed mice without applied nerve injury (*n* = 4 paired observations per time bin; 8 mice total). Error bars represent standard deviation of biological replicates. No significant differences were detected among each of the time intervals post‐implantation, Students t‐test. (i–l) Maximum intensity projections (MIPs) showing long‐term tracking of individual nerve fibers in a Thy1‐YFP mouse via 1P confocal microscopy: (i) Day 1, (j) Day 3, (k) Day 10, and (l) Day 24 after window implantation (1 day post‐mortem). Distinctive features (red and blue arrows) such as brighter, wider, or tapered regions of axons, bends, etc. serve as fiducials to locate the same area across different time points. (i—k) ∼800 nm excitation, 10× objective, FITC channel. (l) 890 nm excitation, 16× objective, acquired on a separate microscope configuration.

To evaluate functional impact, we performed gridwalk testing before and after implantation (*n* = 4) and found no statistically significant differences between windowed and contralateral hindlimbs at any of the sampled time points (*n* = 1 mouse per condition, Figure 1h). To further assess whether using our technique reduces nerve function, we performed survival muscle force testing and/or non‐survival electromyography testing on two of the muscles innervated by the sciatic nerve (gastrocnemius and tibialis anterior) 3 months after nerve window implantation. No deficits in muscle strength were observed (Figure S2), and compound muscle action potential (CMAP) measurements were within the normal range [14] (Table S2). Furthermore, histological analysis of transverse sections of the sciatic nerve ∼90 days post‐implantation revealed no significant differences in axon density or caliber compared to the contralateral nerve (Figure 1f,g and Table S3). In addition, in vivo confocal microscopy through the PDMS window in Thy1‐YFP mice revealed that individual axons could be repeatedly visualized in the same locations over 24 days (Figure 1i–l), indicating preserved spatial organization and enabling longitudinal structural tracking. Individual Thy1‐YFP+ axon diameters measured from high‐resolution confocal images (Figure 2b,c) were 4.0 ± 1.0 µm (*n* = 23 fibers), consistent with values for large, myelinated mouse sciatic nerve axons. Under the imaging conditions used, Thy1‐YFP and S100‐GFP signals did not exhibit photobleaching and remained stable within individual sessions and across repeated imaging over multiple days to weeks, and the same axonal structures could be reliably tracked over time without evidence of cumulative photobleaching or phototoxicity.

**FIGURE 2:**
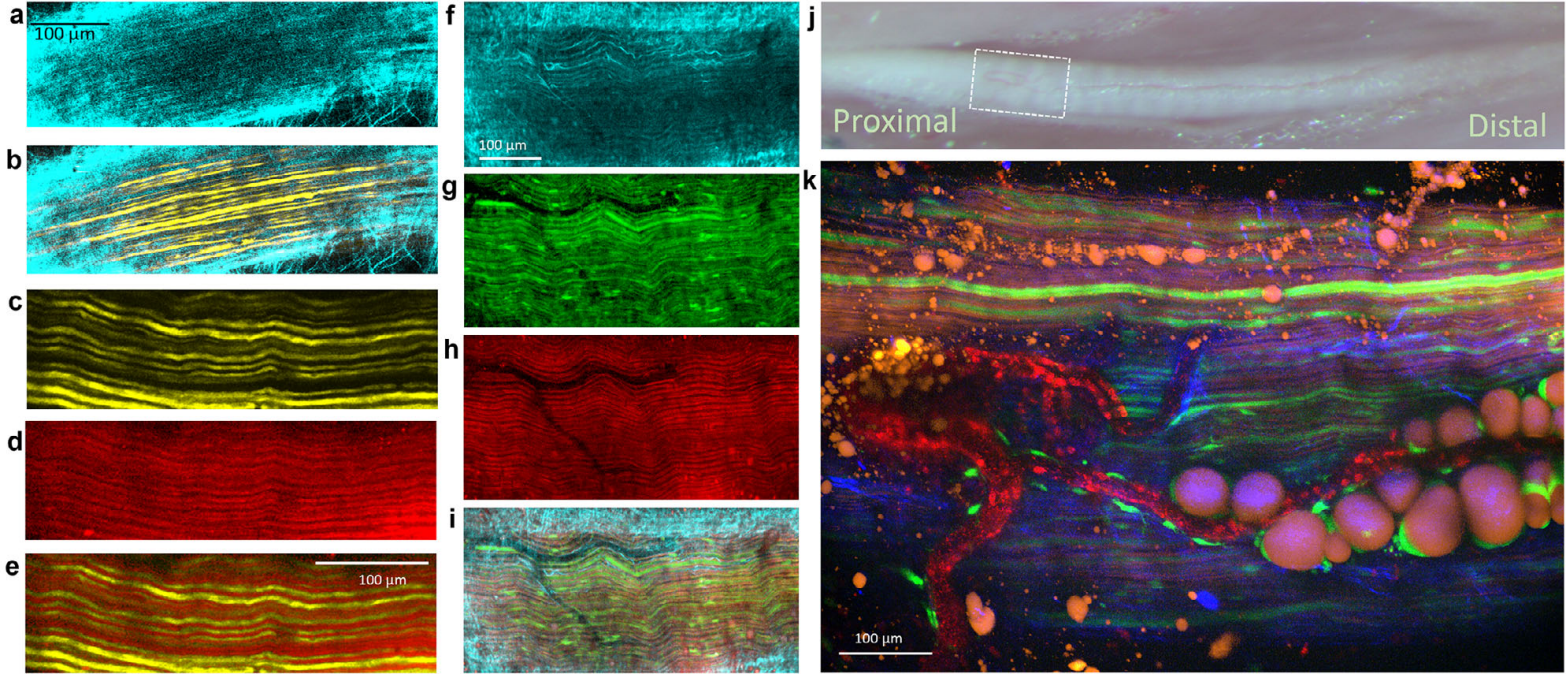
High‐resolution multimodal imaging of peripheral nerve structures. (a,b) SHG imaging of collagen (cyan) in a Thy1‐YFP mouse sciatic nerve, single frame at z = −40 µm (measured from the depth at which the nerve epineurium signal is barely apparent) (b) The same frame including YFP‐labeled axons (yellow). (c–e) 1P confocal imaging of Thy1‐YFP mouse sciatic nerve with Nile Red in situ labeling, MIP of a z‐section (see Video S1). (c) YFP‐labeled axons (yellow). (d) the same frame with only Nile Red labeling myelin shown. (e) Composite of YFP (yellow) and Nile Red (red). (f–i) MIPs (9 µm thick) of an oblique optical section from an S100‐GFP mouse sciatic nerve 1 day after window implantation at depth of ∼50 µm, 2P confocal (see Video S2). (f) SHG imaging of collagen and elastin (cyan). (g) GFP‐labeled SCs (green) (h) Nile Red‐labeled myelin (red) (i) Composite of (f–h). (j) Widefield image of the windowed sciatic nerve in k with a dashed rectangle surrounding the imaged region. (k) Composite MIP combining 1P‐ and 2P‐acquired channels in an S100‐GFP mouse showing GFP + SCs, adipose cells and perivascular cells (green), collagen/elastin (blue; purple where co‐located with myelin), Nile Red‐labeled myelin (orange thin horizontal lines; purple where co‐located with collagen/elastin), collagenous matrix‐embedded lipid globules (brown and lavender), and lipid‐rich material (orange spots across the top and bottom‐left), and Dylight‐649/tomato lectin‐labeled blood vessels (red) (corresponding to Video S3). MIP—maximum intensity projection.

### 2.2 High‐Resolution Multimodal and Dynamic Imaging of Peripheral Nerve Structures

Single‐ (1P) and 2P confocal microscopy enabled rapid, high‐contrast imaging of fluorescently labeled structures, such as axons and SCs, within ∼30 to 100 µm of the nerve surface in transgenic mice. SHG imaging provided intrinsic contrast for non‐fluorescent components such as collagen and elastin within the nerve's ECM as shown in Figure 2a,b and Figure 2f,i,k. Transgenic mice expressing YFP under the Thy1 promoter (predominantly in neurons) or GFP under the S100 promoter (in SCs), along with in situ application of Nile Red and IV administration of fluorophore‐conjugated tomato lectin, enabled clear visualization of axons, SCs, myelin, blood vessels, adipose tissue, and some ECM (Figure 2k). YFP+ axons were identified based on their characteristic continuous, elongated fiber‐like geometry parallel to the nerve axis, with branching and growth‐cone‐like structures visible during regeneration, distinct from mobile round or amoeboid YFP+ cells in the nerve environment [9]. Intact, myelinating SCs in S100‐GFP mice were similarly identified by their characteristic elongated morphology ensheathing axons. We also observed GFP + cells with several other distinct morphologies. These included ∼8 µm round cells and spindle‐shaped cells, predominantly external to the epineurium, consistent with non‐SC GFP + cell types reported for this reporter, including Langerhans cells or macrophages [11]. Beneath the epineurium, we additionally observed thin, elongated GFP+ cells that were sometimes aligned with nerve fibers but distinct from myelinating SCs.

This imaging strategy enabled multiplexed imaging of both cellular and non‐cellular components of peripheral nerves in a comprehensive, multiscale fashion. Since collagen is a natural endogenous contrast agent via SHG [15], we were able to image individual endoneurial channels at cellular‐scale resolution using 2P excitation at 890 nm in conjunction with the DAPI filter in fluorescent (Figure 2a,f) and non‐fluorescent mice (not shown). We also assessed the ability of Nile Red [12] to highlight the myelin and serve as a complementary marker to axon or SC labeling as well as highlight other lipid‐rich species. To do this, we applied Nile Red topically through the injection port of the nerve window in Thy1‐YFP mice (Figure 2c–e and Video S1) and S100‐GFP mice (Figure 2h,i,k and Video S2) between 1 and 34 days after implantation. We observed clear Nile Red labeling of myelin sheaths and lipid globules (Figures 2k and 5b) for both strains and GFP labeling of adipocytes in S100‐GFP mice (Figure 2k). To visualize blood vessels in the nerve, we administered Dylight‐649‐conjugated tomato lectin, which binds to vascular endothelial cells [16] (Figure 2k and Video S3). This also enhanced contrast for some blood cells, enabling dynamic tracking of blood flow (Videos S4 and S5), showing that the window supports visualizing high‐speed physiological processes in vivo.

### 2.3 Visualization of Acute Injury Response Shows SCs Motility and Rapid Loss of GFP/YFP Signal in Injured Fibers

The optical window enabled real‐time imaging of peripheral nerve responses to injury. Within minutes of partial crush injuries in S100‐GFP mice, SCs displayed signs of swelling, retraction, and positional shifts, with qualitatively similar results in both one‐ and two‐crush injuries (Figure 3 and Figure S3). Nile Red and SHG imaging showed that myelin sheaths and endoneurial collagen outside of the acute injury zone were colocalized and remained stationary immediately before and after injury as SCs withdrew. Within 20 to 30 min (Figure 3b–d and Figure S3a), SCs of injured nerve fibers were visibly disrupted with strongly decreased GFP intensity and/or apparently displaced cytoplasm, and both GFP and Nile Red intensity appeared to decrease. Further visual changes among affected SCs after 1 to 2 h included swelling, beading up (Figure 3e), and apparent SC membrane retraction (Figure S3b). Thus, SCs can be seen to undergo dynamic changes, potentially converting to the mobile, repair phenotype [17], in response to mechanical injuries in vivo.

**FIGURE 3:**
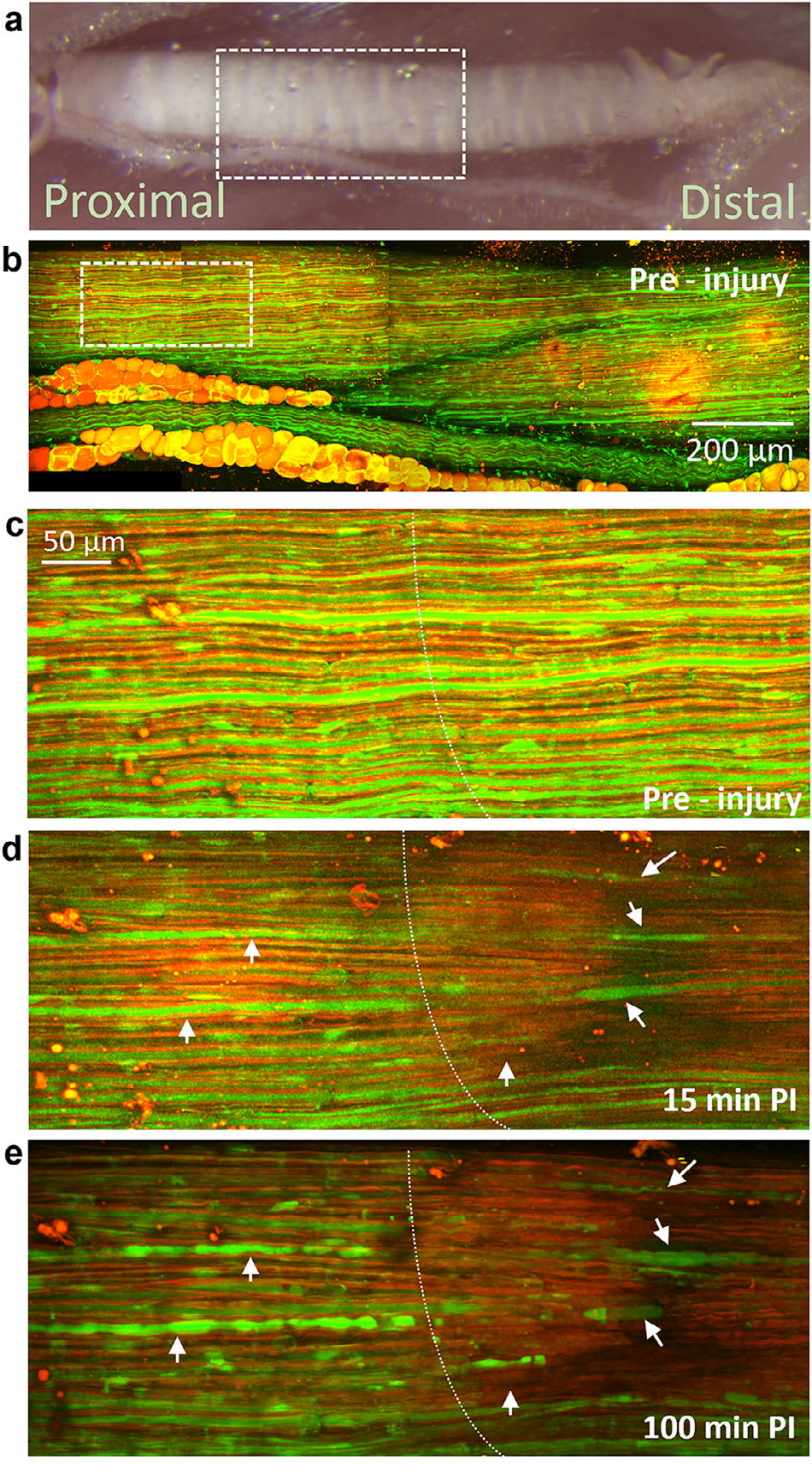
Acute Schwann cells (SCs) response to partial crush injury. (a) Widefield image of the sciatic nerve in a S100‐GFP mouse with the windowed nerve pre‐injury with a dashed rectangle surrounding the imaged region. (b), Mosaic of MIPs of the rectangle outlined in (a). SCs (S100‐GFP) appear green; myelin and lipids appear red due to Nile Red labeling. (c–e) MIPs before and after partial crush injury of the rectangle outlined in (b). Scales in c‐e are marked in (c). Dashed lines indicate approximate injury zone boundaries. (c) Pre‐injury, with intact GFP + SCs aligned along nerve fibers. (d) 15 min post‐injury, showing disrupted injury zones, increased diffuse red signal in some areas, and overall reduced GFP signal from SCs within and near the injury zone. (e) 100 min post‐injury, with further morphological changes in SCs. White arrows mark SCs exhibiting swelling, shape alterations, positional shifts, apparent cytoplasmic retraction, or other structural changes associated with injury.

We performed similar experiments in Thy1‐YFP mice to examine the early stages of nerve regeneration in the visualization of axons. A stretch injury was initially applied to produce a broad zone of axonal disruption across which early regenerative behavior could be observed, followed by partial transection injury to disrupt endoneurial scaffolding, and nerve window implantation (Figure 4a). Approximately 2 h afterward, the regions of the axons that were not physically disrupted appeared intact (Figure 4b), with a clear, well‐defined YFP+ signal throughout the surface of the nerve. After 1–2 days, we observed a significant reduction in the YFP signal in the visible axons distal to the injured region, indicating axonal degeneration, with more complete fragmentation or fading of axons directly impacted by the partial transection (Figure 4c and Figure S4a). After 4 days, thinner, newly sprouted YFP +, growth‐cone‐tipped axonal processes had extended from the proximal nerve segments and penetrated through the injury zone (Figure 4d and Figure S4b), appearing to regrow along the paths of the degenerated axons (Figure 4e,f), reflecting active regeneration. By days 17–22 post‐injury, growth cones or residual growth cone‐like structures remained visible at the tips of axons near the injury zone (Figure S4c,d). Based on the appearance of YFP+ axons at locations where none were previously observed in the partial transection model, we estimate minimum regeneration rates on the order of 1 mm/day or greater, consistent with published values for mice sciatic nerve regeneration ∼1–4 mm/day [18]. Such growth cones have previously been seen only in live non‐mammals (e.g., zebrafish [19]) and in relatively superficial anatomical regions of mice (saphenous [20] and plantar nerves [21]), demonstrating the platform's ability to capture dynamic regenerative responses of axons and vascular structures at subcellular resolution in a major peripheral nerve in a live mammal.

**FIGURE 4:**
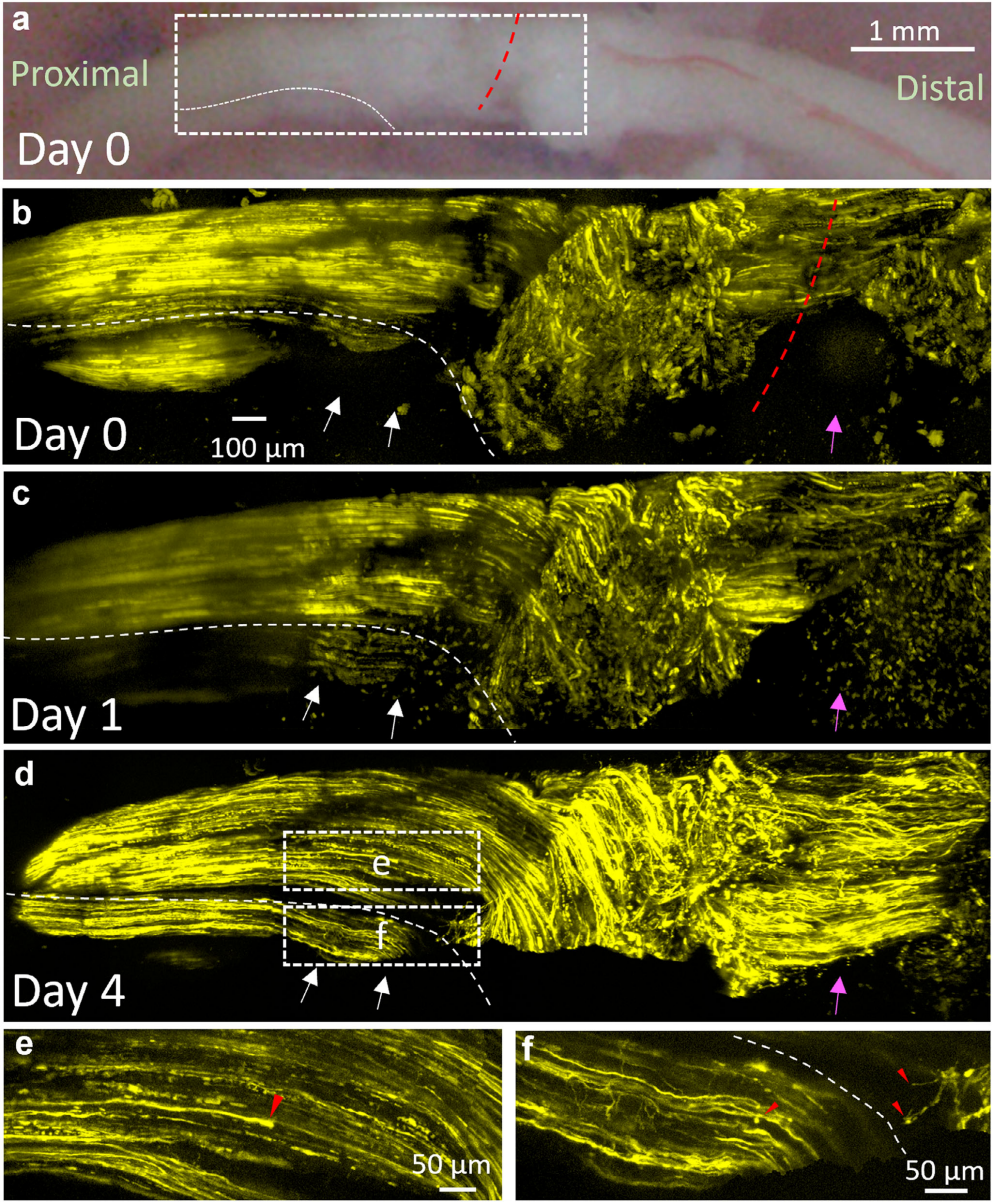
Regeneration‐associated axon morphology and growth cones after nerve transection injury. (a) Widefield image of a sciatic nerve in a Thy1‐YFP mouse <1 day after injury. A white dashed line marks the visible boundary between a large, more superficial fascicle (above the line) which overlies a smaller, deeper fascicle (below). A red dashed line marks the approximate transection path only for the superficial fascicle; the deeper fascicle's transection site is obscured. (b–d) Mosaic MIPs (1P, FITC channel) of the dashed rectangular region in a showing axonal degeneration and regeneration over time. The two fascicles degenerated at different rates, with more advanced degeneration in the deeper fascicle. Proximal nerve fiber ends appear splayed and extruding through the epineurium at the transection site. White and pink arrows indicate regions of degeneration and subsequent regrowth on the deeper and more superficial fascicle, respectively. (b) Day 0: degenerating nerve tissue throughout the injury zone. Red and white dashed lines as in (a). The distal portion of the fascicle on the bottom‐left (white arrows) is not visible in this image due to acquisition depth range limits and scattering. (c) Day 1: axonal degradation and YFP signal loss in damaged fibers (white and pink arrows), with progressive degeneration. (d) Day 4: newly extending axons roughly aligned with day 0 axons appear within previously degenerated regions (white and pink arrows). (e,f) Close‐ups of regenerating axons at day 4 in the regions outlined by the dashed rectangles in (d). Regenerating axons extend along residual extracellular pathways, with multiple enlarged tips (red arrowheads) visible at leading edges of sprouting fibers, suggesting active branching.

### 2.4 Axonal Degeneration and Regrowth in Compression Injury With Localized Epineurial Disruption

To investigate how regenerating axons are influenced by endoneurial pathways, we performed a compression injury (representative example shown in Figure 5a) to simulate a Sunderland Grade II injury with axonal disruption and preserved endoneurial architecture. On the day of injury, 2P imaging showed disrupted axons throughout the nerve, with the most injured fascicle (at bottom‐left in Figure 5b) being virtually depleted of YFP signal while Nile Red marking myelin and SHG marking collagen (SHG not shown separately), remained nearly coextensive, reflecting preserved endoneurial channels (Figure 5b,d and Video S6). In a second trial with a similar injury (Figure S5), the axonal signal was completely depleted by day 2 (Figure S5a).

**FIGURE 5:**
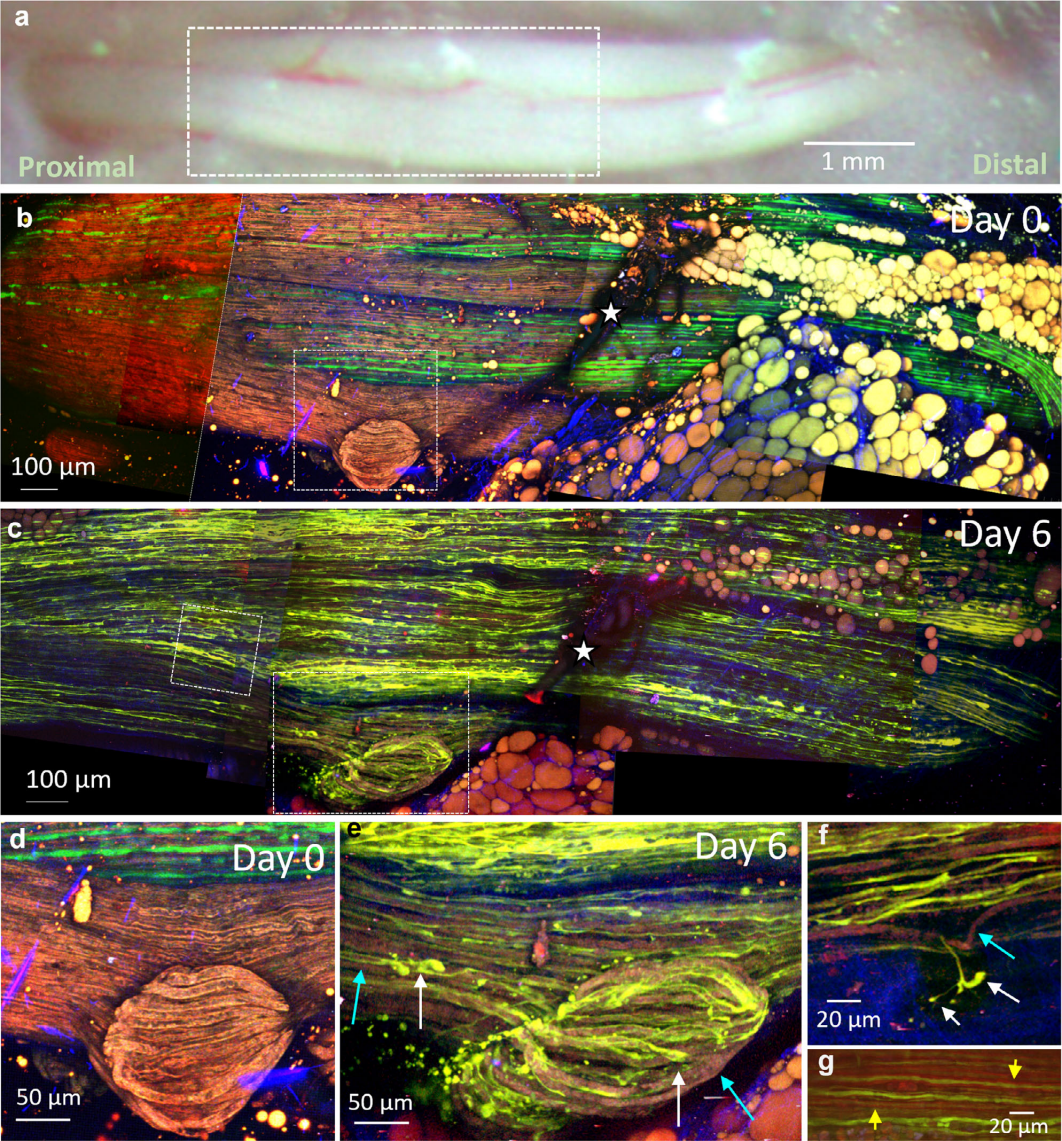
Axonal disruption and regrowth in a Thy1‐YFP mouse sciatic nerve subjected to compound compression injury. (a) Widefield representative example of a compression injury with a dashed rectangle surrounding the rough equivalent of the imaged region. (b) Day 0: Mosaic of MIPs of 1P and 2P z‐stacks (separated by white dashed lined, 1P on the left) on the day of injury, showing YFP (green), collagen (blue in 2P only), and Nile Red (red). Endoneurial channels appear salmon color with 2P imaging due to the close association of myelin and collagen, and lipid droplets appear yellow‐white or salmon. A dashed rectangle surrounding the imaged region in (d). (c) Day 6: Mosaic of 2P z‐stacks of the same nerve region as in (b). Regenerating axons appear yellow (from co‐emission in the TRITC channel) due to fibrotic build‐up leading to increased scattering. A smaller dashed square marks the region shown in (f); a larger dashed rectangle marks the region shown in (e). (d–f), Close‐ups from the regions in dashed white rectangles in (b) and (c). (d), Close‐up of the injured fascicle with extruded endoneurium in (b), showing that axons in the affected fascicle are almost entirely absent compared to axons in the adjacent bundle. (e) Day 6 post‐injury, intact de‐axonated endoneurial channels (cyan arrows) within an injured fascicle and the extruded region, and new actively regenerating axons with growth cone‐like structures (white arrows). (f) Day 6 post‐injury, 16 µm‐thick optical slice of the left rectangle outline in (c) showing a regenerating axon (yellow) that has extended away from a surgically deformed endoneurial channel (red, indicated by cyan arrow) and sprouted multiple axonal growth tips (indicated by white arrows) into a “hole” in the epineural collagen (blue). See also Video S8 for the full z‐stack of this region. (g) Magnified view of a region in this trial at 34 days post‐injury (myelin in red, axons in green), showing regenerated and remyelinated axons (yellow arrows), MIP of 2P image with 730 nm excitation. In (b) and (c), a white star marks a suture on the nerve surface in the epineurium, used as a fiducial.

By day 6, axons had extended along the injury zone, generally following the longitudinal axis of the nerve with a disorganized, meandering pattern (Figure 5c,e and Video S7). Most regenerating axons remained in apparent physical contact with and/or closely aligned with residual endoneurial channels (Figure 5c,e,f), indicating likely tropic guidance [22]. Also, in this trial at day 0, small defects of the epineurium were introduced surgically, causing an extrusion of the underlying endoneurium to protrude out of the nerve (Figure 5b,d) with features suggestive of a neuroma‐in‐continuity [23, 24]. This displaced endoneurial tissue provided a scaffold along which regenerating axons could be tracked. In this case, at day 6 the displaced endoneurium was also partly innervated, with multiple axons partially co‐located with surgically displaced residual endoneurial channels (Figure 5e and Video S8). In addition, at a spot where a suture puncture had caused a ∼30 µm epineural defect/hole and sharp deformation of a residual endoneurial channel, a regenerating axon appeared to deviate from the residual endoneurial channel and extended multiple growth cones into the epineural hole (Figure 5f). In the second compression injury trial (Figure S5), substantial and organized axonal regrowth with a typical striated appearance, along with fine, growth cone‐like processes aligned along the epineurium, were similarly visible by day 17 (Figure S5b,c). These observations illustrate how the endoneurium and epineurium, easily trackable using a peripheral nerve window, guide and disrupt axonal pathfinding even in structurally disrupted tissue.

By 34 days post‐injury, the regenerated axons in this nerve exhibited a post‐regenerative morphology with visible myelination based on Nile Red labeling (Figure 5g) (which was confirmed by histology; not shown). This indicates that not only can axons regenerate after injury with the nerve window model, but that the long‐term course of structural repair and myelination can also be observed.

### 2.5 Visualization of Nerve Repair Across a Conduit in a Transection Model

We next demonstrated how a nerve window technique can be applied to visualize relatively slow biological processes longitudinally in live animals. To observe a delayed regenerative process that spans several weeks, we used a conduit repair model in which the nerve is completely transected, leaving the two ends separated by a gap. During imaging studies across three Thy1‐YFP and 3 S100‐GFP mice followed for 19 to 77 days, tissue cable formation was observed in all six mice, fluorescent regrowth (YFP+ axons or GFP + SCs) was observed in five of the six mice and signs of functional recovery were noted in five of the six mice (Table S4). In S100‐GFP mice, small GFP + cells consistent with immune infiltrates were observed throughout the conduit during regeneration, with elongated GFP + cells consistent with SCs visible at later timepoints. In the representative Thy1‐YFP mouse in Figure 6, beginning with a saline‐filled gap space (Figure 6a), the conduit was progressively populated by mobile YFP+ cells (likely immune cells and/or stromal cells) accumulating within the conduit (Figure 6b) reaching a high density at day 11 (Figure 6c). By day 25, substantial regrowth of endogenously labeled fluorescent axonal sprouts was visible (Figure 6d), with axons appearing bundled and most of them roughly following the axis of the conduit. This organization was maintained and gradually increased in density by day 35 (Figure 6e) and was accompanied by signs of functional recovery, such as grasping ability and sensation (not quantified). Histology of the distal nerve at 59 days (Figure 6f,g) revealed myelinated axons.

**FIGURE 6:**
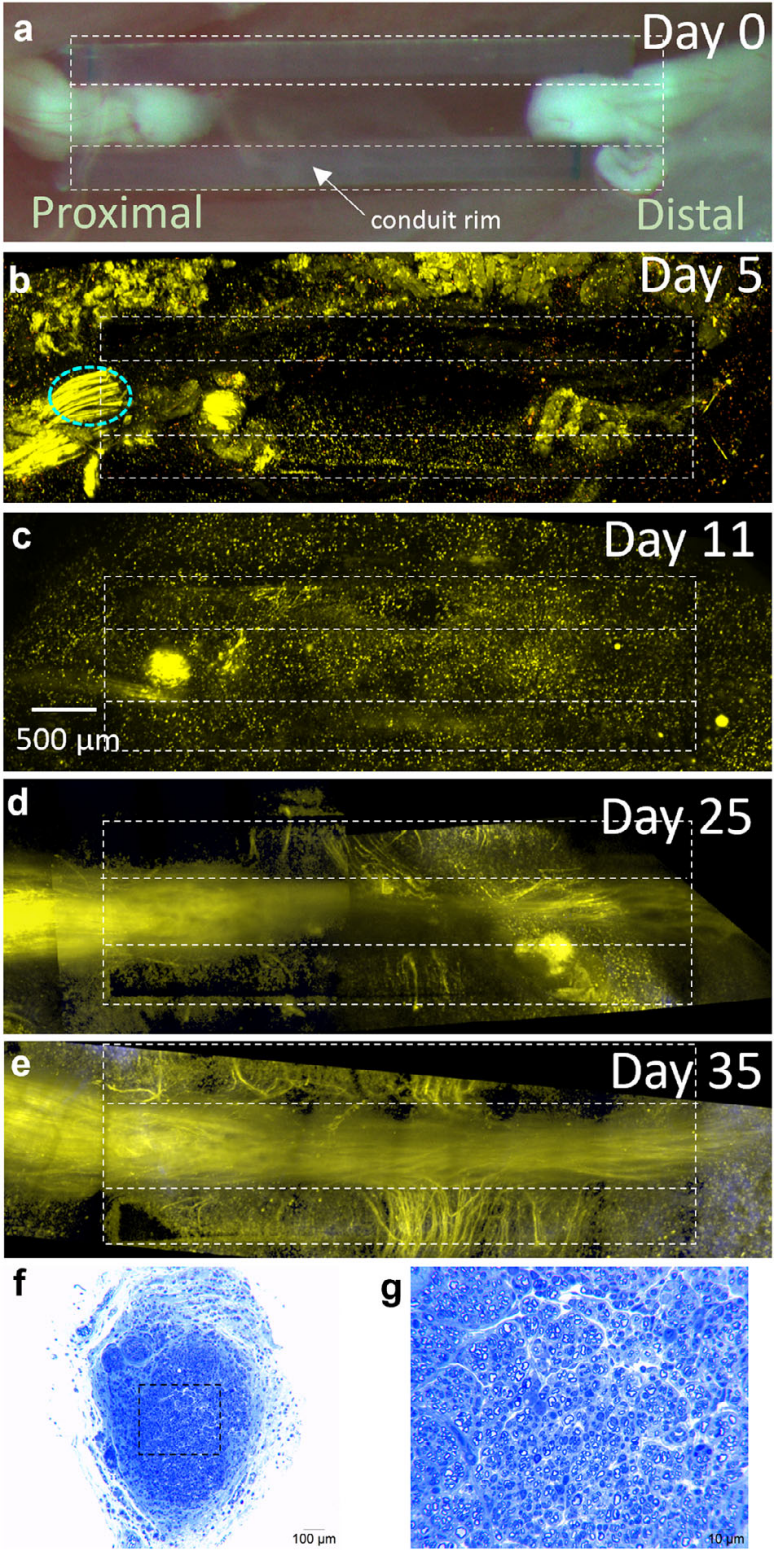
Longitudinal imaging of axonal regeneration in the gap nerve model. Visualization of nerve regrowth across a 2.5 mm gap in a Thy1‐YFP mouse, repaired using a 4 mm PDMS hemitube conduit following complete nerve transection. (a) Widefield representative example of nerve stumps in a repair conduit, day 0. Dashed rectangle highlights the repair conduit. (b–e) 1P confocal microscopy (ex/em 488 nm/FITC) mosaics of MIPs of the nerve conduit. (b) Day 5: limited cell penetration into the saline‐filled gap. The bright fibrous bundle on the left, indicated by a cyan dashed oval, is part of a suture thread that was located above the conduit during this image acquisition. (c) Day 11: significant presence of YFP + mobile cells and some axonal growth. The largest fascicle of the proximal stump shaft is not visible due to Wallerian degeneration and intact fibrotic build‐up obscuring the YFP signal. (d) Day 25: substantial axonal regeneration with bundled fibers aligned along the conduit axis. Nerve fiber regrowth extending to the distal end (e) Day 35: continued increase in axonal density and structural organization. (f,g) Histological section from the distal end of the regrown region of the nerve at day 59 confirms the presence of small, regenerative myelinated axons.

## 3 Discussion

We developed a minimally invasive, stable imaging platform that enables longitudinal, cellular‐resolution optical access to peripheral nerves in vivo for weeks to months. The system is based on a soft, compliant PDMS window secured to the skin above the nerve, which accommodates natural limb motion while maintaining optical clarity and avoiding functional disruption of the nerve. This makes it well‐suited for studying peripheral nerve biology in live mammalian models. Prior approaches to nerve and PNI imaging, such as repeated surgical exposures [25] for 1 to 2 weeks, stiff rubber/glass windows [7], or methods limited to superficial nerves such as the saphenous [20, 26] and plantar nerves [21, 27], have been applied successfully in rodents and in non‐mammalian (e.g., salamander [28], zebrafish [29]) models, but are unsuitable for chronic and longitudinal imaging of non‐superficial nerve trunks over extended timeframes.

Stiff window implants are prone to instability, misalignment, or potential tissue damage, and are also susceptible to grooming behavior in rodents, particularly when located in easily accessible areas like the hind limb. In contrast, PDMS has a combination of mechanical and biological properties ideal for long‐term implantation: its low elastic modulus is comparable to soft tissue, allowing dynamic conformity with minimal mechanical trauma, and its low immunogenicity [30] contributes to implant stability over time. Indeed, PDMS windows have been shown to remain implanted for longer [31] than other non‐bone‐anchored soft‐tissue windows [32], enough for the duration of in vivo nerve regeneration experiments, which can be months [33, 34, 35]. PDMS is also well‐suited for intravital imaging [36] due to its optical properties: it has high optical transparency across a broad spectral range (UV to near‐infrared), low autofluorescence, and minimal light scattering. Its refractive index (∼1.40–1.42 [37]) is close to that of biological tissue, which reduces spherical aberration and enhances image clarity during deep tissue imaging. Furthermore, its excellent biocompatibility tends to limit fibrotic tissue formation, preserving a level of optical clarity over time.

Our platform, combining the PDMS window with confocal, two‐photon, and SHG microscopy, enables high‐resolution, multi‐channel imaging of axons, SCs, myelin, collagen, and blood vessels in the sciatic nerve through genetically encoded fluorescence (Thy1‐YFP, Thy1‐GCaMP, S100‐GFP), exogenous contrast agents (Nile Red, fluorophore‐conjugated tomato lectin), and label‐free imaging (SHG). Because the encoded fluorescent reporters are constitutively expressed, fluorescent signals are continuously replenished between imaging sessions, enabling repeated longitudinal imaging without cumulative signal loss. This multiplexed approach achieves simultaneous visualization of key anatomical and cellular structures across multiple imaging modalities. Using this system, we captured dynamic injury responses at cellular resolution and a variety of dynamic events from the motion of blood cells to complete nerve regeneration. SCs exhibited rapid morphological changes and retraction following crush or compression injuries, indicating possible dedifferentiation, while axonal fragmentation became apparent within 24–48 h. By 4–6 days, regenerating axons with growth cone‐like structures extended along preserved endoneurial pathways. These observations strongly support the notion that the ECM that remains intact during injuries provides structural guidance for axon regrowth. The window technique also allowed us to follow regeneration after complete transection, followed by conduit repair. Axons were observed extending through a PDMS conduit over a 2.5 mm gap, demonstrating that this platform supports studies of both spontaneous and engineered repair.

Despite its advantages, the system is subject to certain limitations. The imaging depth within the nerve was intrinsically limited by tissue scattering, especially in regions of high myelin density, thus the observations reported here largely reflect the superficial‐most estimated 5%–30% of the nerve volume. As a result, degeneration or regeneration across the full nerve cross‐section cannot be directly evaluated. In addition, a fibrotic membrane typically formed against the PDMS window within 2–3 weeks, reducing the contrast and resolution to some degree at all depths. This fibrosis, which results from the animal's immune response [38] and universally accompanies foreign‐body implants [39], complicates imaging of regeneration, especially when newly regrowing axons may be intermixed with fibrotic material. While mechanical clearing of fibrotic tissue through the window port effectively extended the usable imaging period in most cases, we did not rigorously evaluate whether such interventions could disturb healing outcomes. As such, it remains unclear whether fibrotic membrane removal, particularly in regions of active regeneration, may itself disrupt the regenerative process. In addition, although not systematically explored here, our anecdotal observations suggest that more immunocompetent strains may present experimental trade‐offs in terms of fibrosis/imaging clarity and regeneration capacity, which may ultimately require strain‐specific adaptations. Future designs may benefit from anti‐fibrotic coatings, adaptive optics, three‐photon excitation, or longer‐wavelength emitters such as deep red fluorescent proteins or near‐infrared (NIR) contrast agents.

Furthermore, the two main transgenic reporter mouse strains used (Thy1‐YFP and S100‐GFP) are not strictly cell‐type‐specific; besides neurons, other YFP+ cells observed within the Thy1‐YFP mice (e.g., Figure 6b,c) likely represent Thy1‐expressing stromal or fibroblast‐lineage populations [40], and the S100‐GFP reporter also labels non‐Schwann cell populations including Langerhans cells, adipose cells [11], and some perivascular cells (Figure 2k). Additionally, while Nile Red has been used for in vivo lipid imaging without reported adverse effects [38], the potential effects of repeated topically applied Nile Red on nerve regeneration or local inflammation were not formally assessed.

The experiments presented here serve as proof‐of‐concept demonstrations of the feasibility of tracking axonal regeneration across a range of injury severities. However, behavioral and physiological assessments showing no evidence of function impairment in the non‐injury window model were limited in sample size (gridwalk, *n* = 4; muscle force and electromyography, *n* = 1 per condition); these measurements are presented as supplementary observations rather than primary evidence of functional integrity. We also did not directly compare injury outcomes to non‐window controls, or conduct comprehensive functional testing to assess regeneration quality. Additionally, the number of animals with complete longitudinal imaging datasets for each injury model was limited by the practical demands of the imaging protocol, which required frequent anesthesia sessions and manual image acquisition at each timepoint. Thus, several experimental injury groups remain underpowered for definitive quantitative conclusions. Future work will focus on enabling imaging in awake animals through the development of integrated, wearable imaging systems. This will increase temporal resolution and enable imaging through later time points to fully capture the dynamics of SCs and other species over the full regenerative sequence.

For the conduit repair model, the hemitube geometry was chosen over a closed cylindrical conduit to allow better optical access and facilitate tissue integration and nutrient/oxygen exchange for faster nerve regeneration. In this configuration, some regenerating axons were observed along the outer rim of the conduit (Figure 6e), deviating from the main conduit axis. The perpendicular orientation of these escaped fibers may reflect guidance by surface microgrooves produced when the cylindrical tubing was split along its length with a radial cut. Future designs using enclosed or smoother conduit geometries may improve axon confinement.

Motion artifacts remained minimal with proper animal positioning; however, physiological movements such as respiration introduced periodic shifts in focus that limited continuous tracking of small fast‐moving features (Videos S4 and S5), and complete immobilization of the nerve in an uninjured state remains challenging. Additionally, our imaging time points were spaced by days rather than hours, limiting the temporal resolution of some dynamic processes. Increasing imaging frequency, simplifying imaging workflows, incorporating miniaturized imaging hardware (e.g., Wi‐Fi‐enabled fluorescence modules or wearable lens systems), and refining spectral unmixing strategies could enable improved quantitative analysis and reproducibility in future studies.

The major advantage of the nerve window approach is its ability to apply existing and emerging optical systems to visualize the dynamic progression of peripheral nerve events within the same animal across a broad range of temporal scales. This flexibility enables us to dissect causal relationships among events in the nerve regeneration cascade. On longer timescales (days to weeks), processes such as angiogenesis, SCs migration and band of Büngner formation, ECM remodeling, and axonal elongation can be tracked. On shorter timescales (milliseconds to minutes), fast physiological and molecular events, including intraneural blood cell tracking, calcium transients, neuropeptide release, immune cell trafficking and mitochondrial transport, can be captured.

From a translational point of view, the method enables the identification and optimization of pharmaceutical or surgical intervention. It is particularly suited to facilitating and evaluating strategies for biofabricated nerve grafts [41, 42], provided such constructs are sufficiently optically transparent. As such, the technique helps address a critical bottleneck in current nerve repair strategies. The nerve window technique also holds potential for use in models of peripheral neuropathy, where monitoring of ongoing cycles of degeneration and regeneration are essential to understanding disease mechanisms and evaluating novel therapies. It may also serve as a practical tool for assessing surgical repair success early on. For instance, the accumulation or absence of regenerative material within the conduit lumen after gap transection could be directly visualized, helping to identify cases where surgical intervention is unlikely to succeed.

Moreover, this technique creates opportunities to develop new imaging devices such as miniature NIR to shortwave infrared (SWIR)‐range wearable or implant‐embedded cameras to monitor the nerve in awake mice. In the long term, integration with genetically encoded indicators or optogenetic actuators could enable combined sensing and modulation of peripheral nerve activity, with potential applications in basic research and therapeutic development [43]. In combination with the nerve window, such techniques may allow for spatially precise and continuous targeting of neuronal activity in vivo applicable to many applications in peripheral nerve injuries and PNS disorders. Additionally, the use of fiber‐type‐specific Cre driver lines and multi‐reporter models (e.g., Thy1‐CFP/S100‐GFP) could enable identification of specific axon populations and more granular morphometric characterization in future studies.

## 4 Conclusions

Our sciatic‐nerve‐window technique provides a stable, minimally invasive platform for longitudinal, high‐resolution visualization of peripheral nerve cellular behaviors during injury and repair processes. This technique enables repeated imaging of the same nerve region without additional surgical trauma, offering substantial advantages over traditional ex vivo methods. This facilitates detailed characterization of processes such as SC migration, axonal degeneration and regeneration, and guidance along endoneurial channels. The ability to monitor these events in real time opens new avenues for studying the mechanisms underlying peripheral nerve conditions in general and, as demonstrated in this study, the biology of nerve injury and repair. Despite certain technical limitations, this model establishes a versatile and adaptable framework for intravital studies. With further refinements, this technique has strong potential to advance both basic and translational research in peripheral nerve biology.

## 5 Materials and Methods

### 5.1 Animal Models

All procedures were performed under protocols #20180320, #21‐0384 and #24‐029 approved by the Washington University Institutional Animal Care and Use Committee (IACUC).

#### 5.1.1 Pre‐Surgery Preparation

Adult male and female C57BL/6 mice (RRID: IMSR_JAX:000664, JAX: #000664, Jackson Laboratory, Bar Harbor, ME, USA), including Thy1‐YFP (labeling peripheral neurons) [44] (RRID: IMSR_JAX:003782; JAX #003782, JAX Strain: B6.Cg‐Tg(Thy1‐YFP)HJrs/J, Jackson Laboratory), Thy1‐GCaMP (responding to calcium activity in neurons) [10] (RRID:IMSR_JAX:024276; JAX Strain: C57BL/6J‐Tg(Thy1‐GCaMP6f)GP5.5Dkim); JAX #024276, Jackson Laboratory) and B6D2 S100‐GFP (labeling Schwann cells) mice [45] (RRID: IMSR_JAX:005621; JAX #005621; JAX Strain:: B6;D2‐Tg(S100B‐EGFP)1Wjt/J, Jackson Laboratory) weighing 21 to 35 g were used in all procedures. All implanted components were sterilized via autoclaving, UV light exposure, and/or 70% ethanol or isopropanol contact. All fluids for injections or for application during the surgery were pre‐warmed to body temperature. Mice were anesthetized using ∼2% isoflurane and placed on a heating pad to maintain normothermia. The right hindlimb (hip and upper posterior thigh region) was shaved, and residual hair was removed using a depilatory cream (Veet, Reckitt Benckiser, Slough, UK). Care was taken to remove all traces of the depilatory cream with warm water followed by immediate drying. Ophthalmic gel was applied to prevent corneal drying. Mice received subcutaneous injections of buprenorphine (or sustained‐release buprenorphine), carprofen (Rimadyl, Zoetis Inc., Parsippany, NJ, USA), and enrofloxacin (Baytril, Elanco Animal Health, Greenfield, IN, USA), as well as additional fluids for hydration depending on the length of the procedure. The skin on the hindlimb was sanitized with repeated applications of povidone‐iodine solution (Betadine, Avrio Health L.P., Stamford, CT, USA), which was left in place, and a sterile field was established around the planned incision site.

#### 5.1.2 Implants

A variety of hard resin 3D‐printed nerve window frames (Figure S1) were initially custom‐designed using Blender and CAD software to assess approaches for stabilizing the sciatic nerve and supporting regeneration. Window frames were fabricated using stereolithography printing (FormLabs Clear resin) (previously used for cranial implants [46]), rinsed extensively in isopropanol to remove unpolymerized resin, and fitted with a rectangle cut from a #1.5 glass coverslip using a dicing saw, secured with cyanoacrylate glue (Loctite Gel). Each design consisted of a monolithic frame that interlocked with a rigid mouse holder and, in most cases, a recessed saddle to accommodate the nerve. However, the structural rigidity of the material, while crucial for eliminating movement artifacts, introduced unintended injury including nerve compression. Based on these observations, 3D‐printed windows were discontinued in favor of mold‐casted PDMS windows, 18‐mm wide [13] (InfenX, France), which were used as received (pre‐sterilized).

### 5.2 Sciatic Nerve Window Procedure

#### 5.2.1 Nerve Exposure

A stereoscopic surgical microscope was used when operating on the nerve. A straight ∼10–12 mm incision was made longitudinally on the lateral aspect of the hindlimb, directly over the femur or sciatic nerve. Sharp dissection was continued through the skin and the superficial fascia, exposing the muscle. The subcutaneous connective tissue was separated from the underlying muscle surface to create a subcutaneous pocket ∼5 mm beyond the border of the incision to create a space large enough to accommodate the window implant. The intermuscular septum was dissected, and the interval between the vastus lateralis and biceps femoris was divided to expose the sciatic nerve with care taken to not disturb or injure the nerve, including the small branch arising from its lateral aspect.

To securely expose the sciatic nerve without applied nerve injury, the superior aspect of the biceps femoris was secured to the base of the muscle using two to four pieces of 7‐0 or 8‐0 sutures (both resorbable and nonresorbable sutures were used). A small amount of tissue on the biceps femoris edge was sometimes resected. Variations of these procedures, including suture placement, were based on individual anatomy, the location of the skin incision, and the nerve region of interest. In some trials, a PDMS hemitube cut from 1.1 mm i.d. medical tubing (SF Medical Products GmbH, Hudson, MA, USA) was secured beneath a mobilized and/or injured nerve using a single 9‐0 suture to stabilize the nerve and reduce movement artifacts during imaging, although this did not appear to affect the injury response or imaging outcomes.

#### 5.2.2 Injury Models

For injury models, atraumatic separation of the sciatic nerve from the surrounding muscle (neurolysis) was performed after nerve exposure to mobilize the nerve trunk.

*Partial crush*: A single crush (Figure 3) or two closely spaced crushes (to increase the size of the injury zone for imaging; Figure S3) were applied to the anterior surface of the sciatic nerve near the trifurcation using 25G capsulorhexis forceps, each crush for approximately 10 s.

*Compression* (Figure S5): The sciatic nerve was mobilized and subjected to a compression injury by threading a 6‐0 or 7‐0 silk (Perma‐Hand, Ethicon, Inc., Raritan, NJ, USA) or polyglactin 910 Vicryl (Ethicon, Inc., Raritan, NJ, USA) suture from the biceps femoris, passing it beneath the nerve, and through the vastus lateralis in a mattress suture. The suture was then tightened to reapproximate the muscles, pushing a ∼3 mm‐long segment of the nerve outward and preventing it from retracting into the muscle, causing a compression injury (adapted from ref. [7]).

*Compression and partial transection* (Figure 4 and Figure S4): The nerve was subjected to a compression injury as above to induce diffuse axonal degeneration. The common peroneal fascicle was also cut using Vannas micro‐scissors.

*Compression injury with localized epineurial disruption* (Figure 5): The nerve was first subjected to a compression injury as above. Approximately three hours later, after early degenerative changes had begun, a focal defect in the epineurium was created by tying a nylon monofilament 9‐0 suture (AROSurgical Instruments Corp., Newport Beach, CA, USA) to the nerve surface in a knot and pulling it away, producing a small (∼200 µm) breach. This resulted in localized extrusion of endoneurial tissue through the defect, which was then tracked over time.

*Complete transection with conduit repair* (Figure 6 and Table S4): A 4 mm long hemitubular repair conduit was made prior to surgery by bisecting 1.1 mm i.d. PDMS tubing (SF Medical Products GmbH, Hudson, MA, USA) lengthwise with a straight stainless‐steel blade. The sciatic nerve was fully transected, and the proximal and distal stumps were secured to the conduit using nylon monofilament 10‐0 sutures (Ethicon, Inc., Raritan, NJ, USA) with a 2–3 mm gap between the nerve ends when they were inserted. This confined the nerve gap on four sides once the window was inserted (see “Window implantation”) while allowing the nerve to move.

#### 5.2.3 Window Implantation

Following nerve exposure and/or nerve injury, a PDMS window was implanted as previously described [13], taking care to avoid any damage to the nerve. Briefly, the subcutaneous pocket was filled with saline. The implant was bent in half, inserted into the subcutaneous pocket, and unfolded to lay flat on the tissue surface under the skin. The skin opening was stretched into a round shape and the skin edge tucked under the implant's external brim. (For 3D‐printed windows, the skin edge was affixed to the frame using cyanoacrylate glue and/or silk 6‐0 sutures.) The skin tension kept the implant in place and ensured a seal between the implant's circumferential groove and the cut skin edge. The window fluid pocket was then “flushed” with saline: up to 500 µL of saline was introduced via the injection port (septum) on the implant edge using an insulin syringe, then air bubbles and fluidized debris were carefully aspirated. The window was held in place by skin tension against the groove on the window edge, where the skin edge is protected by the upper brim, and remained anchored long‐term through superficial fascia that regenerates in the holes on the edge of the lower (subdermal) brim and adheres to the dermis.

#### 5.2.4 Post‐Implantation Care and Implant Monitoring

Immediately after surgery, mice were placed in pre‐warmed cages and monitored until they recovered from anesthesia. Analgesics and antibiotics (buprenorphine, enrofloxacin, and rimadyl) were administered subcutaneously or via the implant daily for 3 days postoperatively. The implant edge was disinfected using betadine‐soaked cotton swabs, which also served to check for leakage. Mice were housed with access to enrichment materials. We generally followed previously recommended PDMS window maintenance procedures [13] with adaptations specific to our experiments. Mice were examined regularly for infection, leakage, or implant damage; direct manipulation of the skin edge and peripheral groove was minimized. In mice with conduit gap repair, tissue cable formation was evaluated by widefield inspection of the conduit through the nerve window. In some cases (<15% of trials), the skin edge became dislodged from under the window brim without apparent breach of the PDMS–dermis and was usually repaired successfully.

#### 5.2.5 Window Retention Testing

Sciatic nerve windows were implanted in 31 C57Bl6 mice (28 Thy1‐YFP, 3 Thy1‐GCaMP) and 15 B6D2 mice (S100‐GFP). In addition, 16 mice (4 Thy1‐YFP, 7 S100‐GFP, 5 C57Bl6) received rigid 3D‐printed implants (see Table S1). In the PDMS cohorts, 6 C57Bl6 mice and 8 B6D2 mice were removed from the experiment due to planned experimental endpoints (histology, terminal imaging), incidental mortality, or protocol‐related reasons unrelated to window tolerability or performance (Table S1). The retention rate was calculated as the percentage of mice with an intact window at a given post‐operative day out of all mice still in the experiment. Thus, mice removed before window failure were not counted in retention rate calculations for postoperative days after their removal. Windows were considered failed if the tissue–PDMS seal was breached and a repair attempt was not made within one day, or if a repair attempt for a breached seal failed within one day, or if a thick fibrotic or epithelioid‐type membrane prevented nerve visualization and could not be safely removed without dislodging the implant.

#### 5.2.6 Fibrotic Membrane Clearing

Fibrotic membrane generated against the window implant was optionally cleared (without removing the window) prior to confocal microscopy in windows implanted more than 2 to 3 weeks. Mice first received injections of buprenorphine and rimadyl to reduce potential inflammation and discomfort. The window surface was disinfected with betadine and saline, and the injection port was disinfected with 70% ethanol. The fibrotic membrane was gently separated by introducing saline via the injection port using a syringe to create a fluid‐filled working space. The fibrotic membrane was dissected and moved aside in a minimally disruptive way via the injection port using the syringe needle and/or 25G vitrectomy scissors and/or forceps (QWSTitan, China), taking care to not disturb the PDMS/dermis seal. The underlying tissue occasionally showed mild bleeding immediately after but typically healed within a few days.

### 5.3 In Vivo Imaging

#### 5.3.1 Preparation of Animals for Imaging

A mouse with an implanted PDMS window was anesthetized with isoflurane and treated with eye gel, subcutaneous supplementary fluid injections were administered, and the external window surface was cleaned with a Kimwipe wetted with water and/or ethanol. Prior to fluorescence microscopy, the fibrotic membrane was cleared (see “Fibrotic membrane clearing”), and/or dyes were administered (both optional). Nile Red (cat # 19123, Sigma, St, Louis, MO, USA) (100 to 300 µL of 10^−4^ m solution) was injected under the window via the injection port. Vasculature was labeled using DyLight 649‐conjugated tomato lectin (Lycopersicon esculentum lectin [LEL, TL]; cat. # L32472, Thermo Fisher Scientific, Waltham, MA, USA), 1 µg/mL, was injected in the tail vein at a dose of approximately 100 µL per mouse. The sub‐window space was flushed with saline to minimize nonspecific labeling of tissue and the excess fluid was aspirated to bring the nerve closer to the window's inner surface.

To stabilize the tissue for microscopy, we used a custom 3D printed holder. The mouse was placed on its side in a body‐conforming platform made of Flexible resin (Formlabs Inc., Somerville, MA, USA) and anesthesia tubing was taped to the mouse holder. The platform is hinged so that the pitch of the mouse's lower body could be raised or lowered to ensure that window was oriented horizontally. To limit translation and rotation during imaging, the contralateral hindlimb was pulled through a hole into a pocket in the platform and held there with tape. The windowed hindlimb and tail were taped to the platform. A ∼5 mm × 30 mm × 30 mm portion of a handwarmer (Hothands, HeatMax, LLC, Dalton, GA, USA) was cut and inserted into the holder to maintain the mouse's body temperature. To optimize the nerve's lateral exposure and stably adjust the window to the desired position relative to the underlying tissue without putting out‐of‐plane tension on the window, a 3D‐printed stabilizing ring (Flexible resin) was placed around the implant and was taped down at its edges to the platform. A rigid 3D printed cone‐frustum‐shaped component was lowered on to the window such that its circular lip pressed on the edge of implant, further stabilizing it via friction while leaving the imaging area unobstructed. Genteal Tears Severe Lubricant Eye Gel (Alcon Laboratories, Inc., Fort Worth, TX, USA) and/or water was dispensed onto the nerve window surface to serve as an optical immersion fluid. After imaging, the immersion media was cleaned off and the window edge was treated with betadine and the mouse was placed on a warmed surface to recover.

#### 5.3.2 In Vivo 1P and 2P Confocal Microscopy

In most cases, in vivo confocal microscopy was performed on a multiphoton A1R HD25 laser‐scanning confocal microscope (Nikon) with four PMT detectors and laser lines at 405, 488, 561, 633 nm for 1P imaging, a tunable Ti:Sapphire femtosecond laser (Coherent), non‐descanned detectors for 2P fluorescence and SHG imaging, and a 16X water immersion objective (Nikon). Available emission channels were centered at 446, 525, 575 or 595, and 700 nm with cut‐offs of 25 nm in either direction. Image acquisition was performed in 1P mode with excitation at the wavelength(s) corresponding to one or more visible‐light channels or 2P mode with a single excitation at 890 nm. SHG (collagen and elastin), Nile Red, and either GFP or YFP were simultaneously mapped using 2P mode. Z‐stacks were acquired for each “tile” or field of view, spanning from the nerve window surface to the deepest point in the tissue with discernable signal in 1 to 10 µm intervals. Laser power and detector gain were adjusted for each scan to limit saturation, optimize contrast, and minimize risk of thermal damage, in some cases controlled as a function of focus depth (*z*‐coordinate) using NIS‐elements (Nikon). Exceptions: in the case of Figure 5g, a wavelength of 730 nm was used. For Figure 1i–k, we performed 2P imaging using a custom‐built upright multiphoton microscope equipped with a tunable Ti:Sapphire laser (Chameleon Vision II, Coherent Inc., Santa Clara, CA, USA) tuned toapproximately 800 nm) and a 10 × 1.0 NA Olympus water‐immersion objective, with emission collected via non‐descanned detectors. For Figure S4a,b and S5, GFP‐channel images were acquired using an Olympus widefield fluorescence microscope with a GFP/FITC filter set. For Figure S4c, FITC‐channel epifluorescence was performed on the Nikon A1R.

#### 5.3.3 Processing of Confocal Microscopy Images

Raw data z‐stacks were manually processed with NIS‐elements (Nikon) to black‐out frames that were out of place or distorted due to breathing motion to improve the quality of the maximum intensity projections (MIPs) while preserving spatial information. Unless otherwise noted, in vivo nerve images are shown with proximal on the left and distal on the right, and panels from the same trial are MIPs at comparable depths. A false color was applied to each channel, roughly corresponding to a specific labeled species, as described in the images, without attempting to unmix channels. For z‐stack movies shown in Videos S1–S3 and S6–S8, the blacked‐out frames were replaced with interpolated frames for a smoother appearance. Single‐channel and composite images, including MIPs, orthogonal and oblique cross‐sections, 3D renderings, and movies, were created using Imaris (Oxford Instruments), ImageJ/Fiji, and custom Python scripts.

In some cases, fibrotic buildup on the nerve window surface was removed from 3D renderings using oriented cropping planes in Imaris to better visualize the underlying signal. Channel display settings (brightness, contrast, and gamma) were adjusted individually to maximize image clarity. Tile mosaics were assembled either in Imaris 3D View based on microscope XY stage metadata, with lookup tables adjusted separately for each channel to optimize blending or by first creating MIPs and assembling and blending manually in PowerPoint. In composite images, a species, such as YFP under 2P imaging, that emits in both a primary and a secondary channel (e.g., FITC and TRITC) appear as the additive color mix of the channel colors.

#### 5.3.4 Histology and Histomorphometry

Following euthanasia, both windowed and contralateral sciatic nerves were harvested and immediately immersed in 3% glutaraldehyde in 0.1 m phosphate buffer (pH *7.4*) and stored at 4°C for 1–3 days. Samples were rinsed in buffer and post‐fixed in 1% osmium tetroxide for 1 h at room temperature. Tissues were then dehydrated through a graded ethanol series and embedded in epoxy resin (e.g., Araldite 502). Semi‐thin transverse sections (1 µm) were cut using an ultramicrotome and stained with 1% toluidine blue. Images were captured at 400 × magnification using a light microscope. Quantitative histomorphometric analysis was performed using a semi‐automated binary image‐analysis pipeline (Clemex Vision Professional, Clemex Technologies, Longueuil, Quebec, Canada) developed for peripheral nerve [47]. Binary thresholding was applied to generate masks for axon cores and entire fibers (axon + myelin), allowing measurement of axon diameter, fiber diameter, myelin thickness (calculated as half the difference between fiber and axon diameters), and cross‐sectional areas [48]. G‐ratios were computed as the ratio of axon to fiber diameter. Axon counts and densities were calculated for each field. Five non‐overlapping fields were analyzed per nerve, totaling approximately 0.40 mm^2^ per sample.

### 5.4 Behavioral and Physiological Testing

#### 5.4.1 Foot Fault Testing

Mice were placed on an elevated ∼1 by 2‐foot metal mesh grid with ∼3 cm squares. Initially, they were left on the grid for 5 min to acclimate them. For the test, mice were video‐recorded for 5 to 8 min with the camera positioned under the grid. Each foot placement was scored as follows: 1 (clean step—foot placed on the wire with full grip, no slip), 0.75 (foot on wire with slight adjustment or brief instability but no slip), 0.5 (partial slip—foot initially on wire but slipped off under weight), 0.25 (deep slip—foot slipped significantly through grid but partial recovery), or 0 (Complete miss—foot fell through the grid opening). The foot placement success rate was calculated as one minus the average step score, expressed as a percentage, for each foot. testing instances were binned into time intervals based on time since implantation (pre‐implantation, 3 to 6 Days, 8 to 11 Days, 14 to 25 Days, and 26 to 32 Days)

#### 5.4.2 Functional Recovery Assessment

Functional recovery in conduit repair mice was assessed qualitatively by holding the mouse with its hindlimbs suspended and longitudinally pressing a wooden applicator stick (∼2 mm diameter) to the plantar surface of the ipsilateral hindpaw. A grasping or flexion response to the stimulus was scored as a sign of functional recovery. The contralateral (non‐operated) hindpaw was tested in the same manner as a control.

#### 5.4.3 Muscle Force Testing

The mouse was anesthetized and placed on the muscle physiology system (Aurora Scientific Inc., Aurora, ON, Canada). The hindlimb to be tested was secured in a clamp to minimize movement, avoiding clamping the window itself, and the foot was attached to a force transducer via a footplate, with the ankle positioned at approximately 90 degrees. Needle electrodes were inserted near the sciatic nerve through the skin to stimulate the gastrocnemius and tibialis anterior muscles. Electrical stimulation was applied to measure twitch and tetanic force. Peak torque and torque development rate were measured, and the average of three stimulations was calculated.

#### 5.4.4 Muscle Electromyography Testing

For muscle electromyography (EMG) testing, compound muscle action potentials (CMAPs) were recorded at the experimental endpoint to assess neuromuscular function. Mice were anesthetized, and the window implant was removed to expose the sciatic nerve. Electrical stimulation was applied using epineural hook electrodes (34 AWG stainless steel wire, Omnetics Connector Corporation, Minneapolis, MN, USA) connected to an analog stimulus isolator (Model 2200, A‐M Systems, LLC, Sequim, WA, USA) controlled by software (Red Rock Laboratories, St. Louis, MO, USA). The sciatic nerve was stimulated at 0 Hz (single pulse), 10 Hz (three pulses), and 50 Hz (15 pulses) with 1 mA square cathodic pulses. Recorded signals from electrodes inserted into the muscle were band‐pass filtered (1 Hz low‐pass, 5 kHz high‐pass, 60 Hz notch) and amplified 1000‐fold using a two‐channel microelectrode AC amplifier (model 1800, A–M Systems, Inc.). CMAP peak voltages were then averaged over three technical replicates for each frequency condition [49].

#### 5.4.5 Statistical Analysis

All data are presented as mean ± standard deviation (SD) unless otherwise indicated. Sample sizes (n) represent independent biological replicates (individual animals) unless otherwise specified. Gridwalk foot fault testing data were compared between the windowed and contralateral hindlimb using paired two‐tailed t‐tests at each time bin, with an overall paired t‐test and Wilcoxon signed‐rank test across pooled data. For longitudinal measurements within the same animals, repeated‐measures ANOVA or mixed‐effects models were used where appropriate. No data points were excluded unless a technical failure occurred (e.g., imaging artifacts or loss of window integrity), and all exclusions are reported. In imaging studies, the investigators were not blinded to group allocation during experiments; however, quantitative histological analyses were performed blindly to minimize bias. Statistical significance was defined as *p* < 0.05. Exact p‐values are reported in the figures or figure legends where applicable.

### 5.5 AI Use Statement

The authors used Grammarly solely to assist with language editing, grammar correction, and improvement of manuscript readability. No AI tools were used to generate, analyze, or interpret experimental data, produce scientific conclusions, or create figures.

Claude was used to generate Python scripts used for data analysis and plotting, which were reviewed and tested by the authors before use.

### 5.6 The Use of Software Statement

The graphical abstract was created using BioRender (BioRender, Toronto ON, Canada) under an academic license: *Created in BioRender. Berezin, M. (2026) https://BioRender.com/.z6ugwh9*.

Single‐channel and composite images, including MIPs, orthogonal and oblique cross‐sections, 3D renderings, and movies, were created using Imaris 11.0.0 (Oxford Instruments, UK), ImageJ/Fiji v. 1.54 (RRID:SCR_002285), and custom Python 3.14 scripts

The use of the software complies with its licensing terms and any required acknowledgments have been included in the manuscript.

## Author Contributions

Conceptualization, I.L. and M.B.; Methodology, I.L., E.A., R.P., D.B., and M.W.; Software: I.L. and S.Z.; Investigation, I.L., E.A., R.P., D.H., A.A., M.M., S.Z., J.B., E.F., A.C., M.H.; Formal analysis, I.L., M.H., A.C., S.Z., J.B., E.F.; Data curation, I.L.; Writing – original draft preparation, I.L.; Writing – review and editing, all authors; Visualization, I.L., E.A., J.B., and E.F.; Project administration, I.L.; Funding acquisition, M.B.

## Conflicts of Interest

MYB is a consultant for Sarya LLC and Daxor Inc and a founder of HSpeQ LLC. The other authors declare no conflicts of interest.

## Supporting information




**Supporting file 1**: advs76375‐sup‐0001‐SuppMat.docx.

**Supporting file 2**: advs76375‐sup‐0002‐MovieS1.avi.

**Supporting file 3**: advs76375‐sup‐0003‐MovieS2.avi.

**Supporting file 4**: advs76375‐sup‐0004‐MovieS3.avi.

**Supporting File 5**: advs76375‐sup‐0005‐MovieS4.avi.

**Supporting File 6**: advs76375‐sup‐0006‐MovieS5.avi.

**Supporting File 7**: advs76375‐sup‐0007‐MovieS6.avi.

**Supporting File 8**: advs76375‐sup‐0008‐MovieS7.avi.

**Supporting File 9**: advs76375‐sup‐0009‐MovieS8.avi.

## Data Availability

The original. nd2 files are available from the Washington University Depository at https://doi.org/10.17632/b62vrs9x4x. A custom Python code for the gridwalk analysis with the data is located in the Github depository: https://github.com/MikhailBerezin/Gridwalk_analysis.
