# Supplementary material for: Longitudinal Single‐Axon‐Resolution Imaging of Peripheral Nerve Injury Response in Mice Using an Optical Window Implant
Source: Adv Sci (Weinh). 2026 Jun 28;13(53):e76375. doi: 10.1002/advs.76375 (PMC13336950; doi:10.1002/advs.76375)
Supplement: Supplementary file 1 [file ADVS-13-e76375-s006.docx]

# SUPPLEMENTARY MATERIALS

**Longitudinal single-axon-resolution imaging of peripheral nerve injury response in mice using an optical window implant**

Igor D. Luzhansky, PhD^1,2^, Emma Anisman^2^, Ron Perez^1,3^, Sophia Zhang^2^, Morgan Hoffman^2^, Daniel Hunter, PhD^3,4^, Abby Cherian^2^, Jevon Bonner^2^, Emilia Feria^2^, Ahmed Ahmed^2^, Muneeb Malik^2^, Junwei Du, PhD^2^, Leland C. Sudlow, PhD^2^, David M. Brogan MD, MSc^3^, Matthew D. Wood, PhD^4^, Mikhail Y. Berezin, PhD^1,2^

^1^ Institute of Materials Science and Engineering, Washington University in Saint Louis

^2^ Department of Radiology, Washington University in Saint Louis School of Medicine

^3^ Department of Orthopaedic Surgery, Washington University in Saint Louis School of Medicine

^4^ Department of Surgery, Washington University in Saint Louis School of Medicine

* corresponding author: [berezinm@wustl.edu](mailto:berezinm@wustl.edu)

**SUPPLEMENTARY TABLES**

**Table S1.** Summary of the mouse groups, genotypes, implant types, and outcomes shown in **Figure 1e**. Animals removed before window failure (n = 14) were removed for planned experimental endpoints (histology, terminal imaging), incidental mortality, or protocol-related reasons unrelated to device performance.

| **Group** | **Genotype** | **Implant Type** | **# Mice** | **# Removed Before Window Failure (Days)** | **# Remaining in Experiment (Days)** |
| --- | --- | --- | --- | --- | --- |
| **PDMS window** | | | | | |
| 1A | Thy1-YFP (C57Bl6) | PDMS | 28 | 6 (Days 5­­–30) | 22 (up to Day >90) |
| 1B | Thy1-GCaMP (C57Bl6) | PDMS | 3 | 0 (-) | 3 (up to Day >90) |
| 1C | S100-GFP (B6D2) | PDMS | 15 | 8 (Days 4–36) | 7 (up to Day >90) |
| **Plastic window** | | | | | |
| 2A | Thy1-YFP (C57Bl6) | 3D-Printed | 4 | 0 (-) | 4 (up to Day 16) |
| 2B | S100-GFP(B6D2) | 3D-Printed | 7 | 0 (-) | 7 (up to Day 21) |
| 2C | C57Bl6 | 3D-Printed | 5 | 0 (-) | 5 (up to Day 11) |

**Table S2. Peak CMAP** amplitude measurements for a windowed sciatic nerve (n=1) without applied injury.

| **Experiment** | **Stimulation frequency** | **Peak amplitude (mV)** |
| --- | --- | --- |
| Implanted 90 days | 0 Hz | 22.36 ± 0.37 |
|  | 10 Hz | 22.47 ± 0.18 |
|  | 50 Hz | 19.94 ± 0.83 |

**Table S3.** Morphometric comparison of contralateral (L) versus windowed (R) mouse sciatic nerves at 90 days post-implant. Values are mean ± SD of technical replicates (*n* = 2 slices per nerve); Δ (%) is percent change of R relative to L for a single biological replicate (*n* = 1). No gross morphometric differences were observed between sides.

| **Side** | **Density(axons/mm²)** | **Fiber Ø(µm)** | **Axon Ø(µm)** | **G-ratio** |
| --- | --- | --- | --- | --- |
| **L** | 25,900 ± 1,570 | 14.31 ± 0.43 | 4.93 ± 0.14 | 0.665 ± 0.007 |
| **R** | 26,900 ± 3,250 | 13.13 ± 1.14 | 4.86 ± 0.13 | 0.675 ± 0.007 |
| **Δ (%)** | **+3.8** | **−8.3** | **−1.4** | **+1.5** |

**Table S4.** Summary of nerve transection and conduit repair imaging experiments using PDMS hemitube conduits (Thy1-YFP, n = 3; S100-GFP, n = 3). Only mice with windows retained for at least 14 days are included.

| **Strain** | **N** | **Tissue cable** | **Fluorescent regrowth confirmed** | **Functional recovery noted** |
| --- | --- | --- | --- | --- |
| Thy1-YFP | 3 | 3/3 | 3/3 (YFP+ axons) | 2/3 |
| S100-GFP | 3 | 3/3 | 2/3 (GFP+ SCs in gap) | 3/3 |
| **Combined** | **6** | **6/6** | **5/6** | **5/6** |

**SUPPLEMENTARY FIGURES**

**Supplementary Figure S1. 3D-printed windows**. Examples of 3D-printed nerve windows fabricated with Clear resin, used for the curve in **Figure 1e**, viewed from the external (non-tissue) side. **a,** 3D-printed plastic nerve window with the coverslip attached on the tissue side. **b,** A window implant design with holes in the edge for suture attachment, a “saddle” for the nerve, and a rectangular coverslip that fits over the opening and seals it.

**Supplementary Figure S2**. Muscle force measurements from windowed and contralateral (non-windowed) mouse legs without applied injury (n=1 mouse per condition). Data are from two Thy1-YFP mice (one at 41 days post-implantation, another at 41 and 88 days post-implantation), each tested on both the windowed and contralateral hindlimb. Error bars represent SD of 3 technical replicates. One comparison (Maximum Force Rate in Twitch, tibialis anterior) showed a nominally significant difference between limbs within this animal (asterisk, *); all other comparisons were not significant (n.s.).

**Supplementary Figure S3. Acute Schwann cell response to partial crush injury in an adult S100-GFP mouse. a,** 25 minutes post-injury, showing disrupted injury zones (white dashed lines) with greatly reduced GFP intensity from SCs compared to adjacent regions. **b,** 90 minutes post-injury, showing apparent retraction of GFP signal within an endoneurial channel (white arrow).

**Supplementary Figure S4. Axonal degeneration and regrowth following partial transection injury in a 6-month-old female Thy1-YFP mouse. a,** Day 2 post-injury, showing axonal degeneration. **b,** Day 4 post-injury, showing early axonal regrowth with growth cones (red arrowheads) along longitudinal pathways aligned with the original axon orientations. **c,** Day 17 post-injury, close-up of a region approximately located at the yellow dashed outlined region in **b,** showing growth cones at the leading edges of elongating axons near the injury zone (red arrowheads). **d,** Day 22 post-injury, showing largely regrown axons with growth cone-like structures near the injury zone remaining (red arrowhead) (**a** and **b**, GFP-channel fluorescence; **c**, GFP-channel epifluorescence; **d**, two-photon – green YFP, blue SHG; vertical red dashed lines in **a**, **b**, and **d** are in approximately the same horizontal position to serve as a visual guide. Sciatic nerve is oriented horizontally in all images, with the proximal end toward the left.)

**Supplementary Figure S5. Axonal degeneration and regrowth following compression injury in a 4-month-old female Thy1-YFP mouse. a,** Day 2 post-injury, YFP signal is largely absent except for a single axon (cyan arrowhead). **b,** Day 17, nerve has regained a typical striated appearance from substantial visible axonal regrowth. **c,** Close-up of outlined region in **b** showing regenerating axons with fine growth cone-like processes (red arrowheads) growing along the plane of the epineurium. (GFP-channel fluorescence images. Sciatic nerve is oriented horizontally in all images, with the proximal end toward the left.)
